# Assessing the inner setting among Massachusetts community health centers: opportunities for multilevel investigation and expansion of influences on health equity

**DOI:** 10.1186/s43058-025-00724-x

**Published:** 2025-04-10

**Authors:** Rebekka M. Lee, James G. Daly, Daniel A. Gundersen, Ruth I. Lederman, Susan Dargon-Hart, Jonathan P. Winickoff, Karen M. Emmons, the ISCCCE Partnership

**Affiliations:** 1 Department of Social and Behavioral Sciences, Harvard T.H. Chan School of Public Health, 677 Huntington Avenue 7th Floor, Boston, MA 02115 USA; 2 Rutgers Robert Wood Johnson Medical School, New Brunswick, NJ USA; 3 Dana Farber Cancer Institute, Boston, MA USA; 4 Massachusetts League of Community Health Centers, Boston, MA USA; 5 Massachusetts General Brigham, Harvard Medical School, Boston, MA USA; 6 Implementation Science Center for Cancer Control Equity, Harvard T.H. Chan School of Public Health, Boston, MA USA

**Keywords:** Inner setting, Equity, Community health centers, Context

## Abstract

**Background:**

Implementation science increasingly aims to improve health outcomes in delivery of evidence-based interventions. It is important to understand the inner setting of organizations where interventions are put into place, as setting characteristics can have significant impact on implementation outcomes. Community health centers are increasingly engaged in efforts to improve use of evidence-based cancer control interventions. Taking a comprehensive, partnered approach to measuring the inner setting among a network of community health centers engaged in implementation research ensures assessment of the variability among sites.

**Methods:**

We conducted a cross-sectional survey among staff (*n* = 63) purposively sampled from 12 community health centers in Massachusetts engaged in research at the Implementation Science Center for Cancer Control Equity. The survey assessed inner setting constructs from the Consolidated Framework for Implementation Research, including learning climate, leadership engagement, available resources, and implementation demands/stress using validated measures (Likert scale range: 1 “strongly disagree” to 5 “strongly agree”). Additional equity-focused inner setting items included structural characteristics of the work infrastructure and language access services. Descriptive statistics examined differences by staff role and health center.

**Results:**

Staff rated learning climate (mean = 3.98) and leadership engagement (mean = 3.67) positively, while available resources (mean = 2.78) had the lowest rating, particularly staffing resources. Clinical staff rated the inner context lowest compared to other roles. All centers reported supportive human resource benefits for caregiving and 92% provided tuition assistance, while fewer offered formal mentorship (50%) or affinity groups (33%). Community health centers reported written materials are routinely provided to patients in languages other than English and interpreter services were most common in Spanish, Vietnamese, and Portuguese.

**Conclusions:**

This study provides an assessment of the inner setting within Massachusetts community health centers at the start of a new research collaboration. Periodic follow-up surveys will monitor changes over time. Data can be used in future analyses to explore how inner setting characteristics influence implementation outcomes and impact equitable translation of evidence-based interventions into practice.

Contributions to the literature

- Implementation science increasingly aims to improve equity in delivery of evidence-based interventions; this paper expands the conceptualization of the inner setting.
- A survey was fielded among diverse health center staff, using validated measures of learning climate, leadership engagement, resources, and implementation demands and stress, plus equity-focused items about staff tuition assistance, benefits, and translation services.
- Findings showed variability in responses between and within health centers. Clinical staff rated the health center inner setting lowest.
- The study provides a roadmap for how researchers can collaborate with community partners to collect centralized contextual data that can be applied across studies.

## Background

Clinical research efforts have been increasingly focused on enhancing translation of scientific evidence to maximize the beneficial impact on all populations and address persistent health inequities. In particular, implementation science has focused on identifying the best approaches to deliver effective interventions into clinical practice. Implementation science theories, models, and frameworks emphasize the importance of specifying contextual influences on implementation [1–3]. Accounting for context can help elucidate how or why implementation outcomes are achieved and enhance generalizability [4]. Knowledge about context may inform outcome variation in a specific setting over time as well as across different settings.

The Consolidated Framework for Implementation Research (CFIR) defines the inner setting as the organization where an intervention is implemented, which in the case of this project is the community health center [5]. Inner setting constructs include the organization’s structural characteristics (e.g., size, space), culture (i.e., the organizations’ norms and values), relative priority of the intervention compared to other internal initiatives, alignment with organizational mission and goals, and available resources [5]. Allen and colleagues conducted a systematic review of the organizational characteristics that are associated with implementation outcomes guided by the CFIR inner setting characteristics [6]. Of the 76 studies that met inclusion criteria, there was wide variation in “conceptual and operational definitions of organizational constructs”. Since this review was published, there has been considerable effort to identify a set of organizational measures with sound psychometric properties that could be used to provide some standardization of implementation measures across studies [7–10]. Fernandez and colleagues [8] identified measures for seven CFIR inner setting constructs and adapted them for use in the health center setting.

As implementation science increasingly aims to contribute to efforts to improve equity in access to evidence-based practices and policies [11, 12], it is particularly important to measure aspects of the inner contextual setting in ways that can allow us to generalize knowledge across settings and studies. Furthermore, it is critical to characterize specific aspects of the inner setting that may address equity beyond those typically measured within implementation science. The U.S. Department of Health and Human Services Office of Minority Health set national standards and guidelines for taking an organizational systems approach to integrating culturally and linguistically appropriate services within healthcare organizations [13]. Their framework includes structural characteristics of the work infrastructure [5] such as human resources practices and diversity training as well as language access services [13] that may influence equitable implementation of and access to evidence-based interventions. We have integrated validated measures of these constructs into our study of the inner setting, as they are particularly important to measure in settings like community health centers that predominately serve racially diverse, low-income populations. The aim of this paper is to describe the variability in inner settings across a network of community health centers in Massachusetts that are participating in implementation research projects of the Implementation Science Center for Cancer Control Equity.

## Methods

### Design and setting

The Implementation Science Center for Cancer Control Equity (ISCCCE) is a collaboration between the Massachusetts League of Community Health Centers (Mass League), the Harvard T.H. Chan School of Public Health (HSPH), Massachusetts General Hospital (MGH), and Dana Farber Cancer Institute (DFCI) funded by the National Cancer Institute. Mass League is the state primary care association, which “serves as an information source on community-based health care to policymakers, opinion leaders, and the media, and provides a wide range of technical assistance to health centers and communities” [14]. Mass League collaborates with the ISCCCE Implementation Lab (I-Lab) to build the research capacity of community health centers and supports implementation of evidence-based interventions among community health centers participating in implementation research pilots [15].

This study uses a cross-sectional survey to measure the inner setting of all community health centers that were engaged with ISCCCE when it was initially launched. This includes staff perspectives from: 1) all sites that received funding for and participated in the first round of implementation pilot studies *and* a learning community; and 2) all sites that received funding for and participated in a learning community. The learning community is designed to increase engagement in and organizational capacity for participating in implementation research [15]. Health centers were given the autonomy to express interest in participating in pilots and the learning community. Mass League hosted webinars open to all Massachusetts federally qualified health centers to explain the research opportunities and provide an open forum for questions and answers. Then interested health centers applied through a simple, streamlined process. Informed by CFIR, items on the survey include aspects of the inner setting as well as characteristics of the individual staff responsible for implementation of evidence-based interventions [5].

### Participants and recruitment

ISCCCE research staff collaborated with leaders from Mass League to invite 123 staff members from 12 community health centers to participate in the survey. To gather a range of perspectives on the inner setting of each community health center, 1–3 people were sampled within each of five job type categories – leadership, clinical, quality improvement, community direct service, community outreach and engagement. We included staff who were actively engaged in pilot research and/or Learning Community activities. At community health centers where staff with specific roles were not engaged in ISCCCE activities, the primary contact was asked to provide a roster of staff names in each role. For roles with more than three staff members, study personnel randomly selected participants in each role type using a random number generator.

Once participants were sampled, a Mass League leader sent a prenotification email to all potential participants to notify them about an upcoming survey invitation coming from the ISCCCE research team. Several days after the email notification, health center staff were formally invited to participate in the survey using an individualized link to mitigate duplicate responses. This tiered outreach approach in collaboration with Mass League was designed to build familiarity and trust with the survey among health center staff. Up to three survey reminders were emailed over several weeks. Staff received a $25 gift card for completing the survey.

### Measures

The research team fielded an online survey via REDCap between November 2020 and March 2021. To minimize the respondent burden on community health center staff, the research team and Mass League partners used a collaborative process to review and prioritize items for inclusion. The survey employed full validated scales with good internal consistency and discriminant validity from the implementation science literature on learning climate, available resources, implementation stress, and leadership engagement [8]. All inner setting survey items were measured using a 5-point Likert scale with a “1” rating meaning “strongly disagree” and “5” rating meaning “strongly agree” and had previously been adapted to the community health center setting by Fernandez and colleagues [8]. Four items on learning climate (Cronbach’s alpha = 0.85) defined as “a climate in which leaders express their own fallibility and need for team members’ assistance and input, team members feel that they are essential, valued, and knowledgeable partners in the change process, individuals feel psychologically safe to try new methods, and there is sufficient time and space for reflective thinking” were adopted from the Practice Adaptive Research [16]. Four items assessing the commitment, involvement, and accountability of leaders were adopted from this same measure (Cronbach’s alpha = 0.92) [16]. Three items on general (not intervention-specific) available resources (e.g., money, training, staffing) were from the Organizational Readiness to Change Assessment [17]. Four items on perceived stress, strain, and role overload are from the TCU Organizational Readiness for Change measure (Cronbach’s alpha = 0.85) [18]. Additionally, the survey included items on participants’ roles (i.e., select all that apply from 10 options, see Table 1), years of tenure within the center, and demographics (e.g., gender, race/ethnicity, age).

**Table 1:** Descriptive statistics of community health center staff (*N* = 63) across 12 MA CHCs

	N	%
**Gender**		
Women	49	90.7
Men	5	9.3
**Race/ethnicity**		
White	32	50.8
Black/African American	10	15.9
Asian	9	14.3
Hispanic/Latino	5	7.9
Other	2	3.2
**Role**		
Clinical services	30	47.6
Quality Improvement	27	42.9
Management	25	39.7
Leadership	22	34.9
Administrative	14	22.2
Community outreach	11	17.5
Technology/Data services	8	12.7
Community direct services	7	11.1
Referrals	1	1.6
Consultation	1	1.6
	Mean (SD)	Range
**Age**	31.0 (9.7)	27–63
**Job Tenure**		
Total years in position	4.8 (5.8)	0–27
Total years employed at center	6.3 (6.1)	1–27

We broadly operationalized health equity as ensuring that all health center patients can attain their best possible health, taking intentional consideration of the contextual impacts on health. This means patients with different lived experiences and barriers to care can access services. Examples include providing transportation to those without a car and digital navigation for patients to participate in virtual appointments. We also worked with partners at the Mass League to identify unique characteristics of the community health center inner setting that may influence equity – the main focus of our center. Structural characteristics of the work infrastructure and language access services were identified as top priorities. Dichotomous (yes/no) items on human resource benefits offered by the center (e.g., formal mentoring, tuition assistance, personal counseling or employee assistance programs), and translation services from the Cultural Competency Assessment Tool for Hospitals [13] were also included on surveys of in order to capture aspects of the community health center infrastructure that could support equity and potentially reduce staff turnover, which can create significant challenges for implementation. The survey took an average of 15 min to complete.

### Statistical analysis

Sample demographics are characterized with relative frequencies. Respondent reports of inner setting characteristics are described through means, standard deviations (SD), and intraclass correlation coefficient (ICC) for each item and aggregate scale scores. Together, these provide a description for each item and scale score of (a) average or expected score (mean), (b) the total variability (SD), and (c) the within-CHC variability. The ICC provides a description of to what extent the scale scores are similar or different within CHCs. This is particularly useful for understanding the utility of these measurement tools for analysis of differences among community health centers (or other clusters). For example, larger ICCs indicate there a meaningful share of the variability is due to differences between community health centers, lending the measure to examine differences across CHCs. In contrast, low ICCs indicate there is little variability between CHCs but variability among individuals is still feasible. Moreover, the ICC can be interpreted as the extent to which CHC staff have consistent perceptions of the CHC’s inner setting characteristics, which may be substantively informative in and of itself. Further, we also report frequencies for each response option. Aggregate scores for inner setting characteristics were created by averaging survey responses pertaining to each characteristic as recommended [8].

Stratified analyses describe perception of inner setting characteristics by role. In order to examine the relationship between roles and inner setting characteristics, a non-overlapping role variable was created. Anyone who identified leadership as one of their roles was categorized as a leader. Subsequent role categories created were those who did any clinical work, those who were involved in community direct service or community outreach, and those involved in quality improvement. Any remaining staff were categorized as “other”.

Equity-focused inner setting characteristics are described with percentages. To minimize respondent burden, survey items on HR benefits and translational services available were only included in surveys of those who reported they were involved in management or quality improvement. Participants within a health center did not consistently report health center resources such as tuition reimbursement, languages available, or the existence of a written translation policies. In analysis, if anyone at the center reported these characteristics, that center was counted as having the policy or practice.

## Results

### Respondents

Sixty-three (51.2%) staff members from 12 (100%) MA community health centers completed the survey (Table 1). An average of five staff members (range 2–8) completed the survey at each community health center. Over 90% of staff respondents identified as women. Fifty-one percent identified as white, followed by black/African American (16%) and Asian (14%). The ages of respondents ranged from 27 to 63 years. Respondents reported a wide range of experience with between 0 and 27 years in their current position (mean 5 years). In alignment with the sampling plan design, respondents were in a range of roles at the community health center: 48% reported working in clinical services, 43% in quality improvement, 35% in leadership, 17.5% in community outreach, and 11% in direct community service.

### Inner setting characteristics

Table 2 presents data on commonly measured aspects of the inner setting. Learning climate was found to be high and skewed toward higher scores, with an average score across participating community health centers of 3.98 out of 5. Eighty percent or more of respondents agreed or strongly agreed that “the community health center encourages everyone to share ideas” and “we regularly take time to consider ways to improve how we do things”. Leadership engagement was also found to be strong, with an average score of 3.67 out of 5. Conversely, the summary score for available resources was 2.78 out of 5: 40% of respondents disagree with having enough money and training, 58% disagree with having enough staff. Implementation demands and stress were high on 3 of the 4 measures: 39% reported they were “under too many pressures to do my job effectively”, 60% reported staff members often show signs of stress and strain, 51% reported “the heavy workload reduces program effectiveness”, and 52% reported staff frustration is common at their community health center.

**Table 2:** Perceptions of the CHC inner setting among 63 community health center staff

	**Mean (SD)**	**ICC**
**Learning climate**	**3.98 (0.69)**	**0.004**
We regularly take time to consider ways to improve how we do things	4.12 (0.83)	0.000
This community health center encourages everyone to share ideas	4.05 (0.81)	0.000
This community health center learns from its mistakes	3.78 (0.80)	0.124
When we experience a problem in the community health center, we make a serious effort to figure out what's really going on	3.95 (0.85)	0.041
**Available Resources**	**2.78 (0.90)**	**0.046**
We have the necessary support in terms of budget or financial resources	2.85 (0.99)	0.175
We have the necessary support in terms of training	2.97 (1.07)	0.000
We have the necessary support in terms of staffing	2.52 (1.03)	0.000
**Implementation demands/stress**	**3.35 (0.97)**	**0.068**
I am under too many pressures to do my job effectively	3.00 (1.07)	0.074
Staff members often show signs of stress and strain	3.56 (1.05)	0.070
The heavy workload here reduces program effectiveness	3.35 (1.08)	0.000
Staff frustration is common here	3.47 (1.10)	0.061
**Leadership**	**3.67 (0.80)**	**0.076**
The community health center leadership makes sure that we have the time and space necessary to discuss changes to improve care	3.56 (0.87)	0.006
Leadership in this community health center creates an environment where things can be accomplished	3.65 (0.94)	0.036
Community health center leadership promotes an environment that is an enjoyable place to work	3.65 (0.92)	0.033
Leadership strongly supports community health center change efforts	3.82 (0.86)	0.178

1—Strongly Disagree, 2—Disagree, 3—Neutral, 4—Agree, and 5—Strongly Agree

Standard deviations for aggregate scores indicate there was notable variability in responses. Moreover, the ICC suggests the variability is mostly due to within-CHC differences. Results stratified by role reveal differences in perceptions of the inner setting. Compared to the overall average, clinical staff (*N* = 18) rated learning climate 3.74 (vs. 3.98 average), available resources 2.63 (vs. 2.78 average), and leadership 3.33 (vs. 3.67) lowest. Community-facing roles (*N* = 10) rated learning climate (4.15), available resources (3.03), and leadership highest (3.86). Stress and demands were rated highest among health center leaders (3.63 vs average 3.35).

### Equity-focused inner setting characteristics

We sought to assess structural characteristics of the community health center inner setting expected to address equity among staff and patients. In terms of work infrastructure, most community health centers had management training (75%) and tuition assistance or tuition reimbursement for ongoing education (92%) to support continued learning. HR benefits such as work/life balance programs such as flextime, job sharing or telecommuting (92%) were common at community health centers, as were child or elder care, personal counseling or employee assistance programs (100%). Flexible benefits such as domestic partner benefits, family illness, death, and personal leave policies that accommodate alternative definitions of family were consistently available (100%). Fewer community health centers had formal mentor programs (50%) or affinity groups for racial/ethnic minority staff (33%). Most community health centers reported that written materials are routinely provided to patients in languages other than English, but availability of translation services varied by type: 83% of centers provided translation for patient advance directives and end of visit summaries, 92% for medical instructions and informed consent statements, and 100% for health education materials. Interpreter services were reported for 18 languages, the most common being Spanish, Vietnamese, and Portuguese.

## Discussion

This paper describes the inner contextual environments among a network of Massachusetts community health centers engaged in implementation research. The data are particularly useful in documenting the inner setting during a time of emergency, as these data were collected during the COVID- 19 pandemic when there was a major upheaval to community health center workforce and operations. By utilizing full validated scales aligned with CFIR, we can compare these data will other studies within and outside of ISCCCE. For instance, compared to estimates from health centers in the original validation study, we found that aggregate scores for available resources were lower and implementation demands/stress were higher in this sample of sites [8]. Conversely, learning climate and leadership support scores were slightly higher [8]. This survey data complements other approaches to measure the health center context such as using administrative data to understand the impact of staffing ratios and electronic health record usage on cancer screening rates [19].

The variability we found in some parameters will be quite useful in subsequent analyses where we explore if and how inner setting characteristics influence implementation outcomes in specific studies and across studies. Results indicate that the most variability in this sample was between people within the center, rather than between centers. This finding highlights the value of our sampling approach developed with input from the Mass League, which intentionally included a wide range of perspectives within each organization: primary care providers, nurses, medical assistants, leaders, qualitative improvement staff, and community health workers. Other studies may find less variability if they collect data from just leaders or primary care providers. This is quite important, as different staff will have different levels of participation in implementation efforts, and thus evaluating inner setting at only one staffing level may not fully represent the setting and thus lead to approaches that may be insufficient.

Following our center’s commitment to ensuring that evidence-based interventions reach all those served by the community health centers, we identified existing measures of HR practices and translation and interpreter services that allowed us to expand the conceptualization and measurement of the health center inner setting. Most community health centers had human resource benefits for staff that could contribute to a more equitable workplace (e.g. tuition assistance, employee assistance programs). However, only one third reported affinity groups for staff from different population groups that would be indicative of a culturally inclusive work environment. Most community health centers provided translation of written materials as well as verbal interpreter services (most commonly Spanish, Vietnamese, and Portuguese), which are structural characteristics of the inner setting that promote more equitable access to evidence-based interventions. Future studies could build on these findings to identify how human resource benefits translate into the patient care experience and expand to include other structural characteristics that would address equitable access, such as disability policies and practices that ensure plain language materials.

Following principles of community engaged research [15], the success of this cross-center inner context survey can be largely attributed to our partnership with Mass League. Engaging their team in the design of the survey and outreach activities helped to ensure trust and buy in from community health center participants. The inner setting data collected currently lives in a data ecosystem to enable use across all center studies using a REDCap data request form available on our website [20]. Recently, early-stage investigators have harnessed this data for exploration of the inner setting on rates of colorectal cancer screening [21] as well as utilized as part of preliminary data in training grant proposals. In addition to this survey, we conducted a brief pulse survey of dynamic inner setting constructs in year three of the center and are fielding the full survey again in the final grant year to capture shifts over time in the health center inner setting.

This study is not without limitations. While we were able to capture staff from all community health centers engaged in the initial research pilots and learning community activities, only about half of the staff invited participated in the survey. As with all surveys, non-response may bias estimates. The magnitude of the bias depends on the magnitude of non-response and the differences between respondents and non-respondents. Non-response bias may manifest itself both at individual level estimates as well as community health center estimates. For example, if the non-respondents are different from respondents, and respondents within health centers answered homogenously to an item, the ICCs may be underestimated.

This is perhaps not unexpected, given that this level of response is fairly typical among surveys of the health center workforce and the survey was fielded in the height of the COVID- 19 pandemic. This limited the representativeness of our sample and our power to formally test for differences across roles and sites. We were also limited in the number of constructs within the CFIR-defined inner setting that we could feasibly measure, although we selected those constructs that we felt would be most important in examining implementation outcomes. External validity is also limited: these data are not intended to generalize beyond Massachusetts health centers. Finally, due to the Center-wide focus of this study surveying health center staff participating in a variety of research pilots, we used three items on general resources (versus the full scale that included three items on intervention-specific resources).

## Conclusions

This paper presents a centralized assessment of our health center collaborators’ inner setting, which will support more systematic assessment of the inner setting in our on-going pilots and allow us to evaluate areas across the partnership where efforts could be addressed to improve the inner setting for implementation. We have also developed a separate measure of the outer setting, reported elsewhere [22], with the goal of being able to comprehensively evaluate the impact of context on implementation activities. We believe that a comprehensive assessment of the implementation setting will greatly facilitate our learning from implementation studies and provide new knowledge for the impact of setting on equitable translation of evidence-based practices.

## Data Availability

The data collected and analyzed during the current study are available from the corresponding author on request and will be shared in accordance with Cancer Moonshot funding policies.

## Abbreviations

CFIR: Consolidated Framework for Implementation Research
CHC: Community health center
DFCI: Dana Farber Cancer Institute
HR: Human resources
HSPH: Harvard T.H. Chan School of Public Health
ICC: Intraclass correlation coefficient
I-lab: Implementation Lab
ISCCCE: Implementation Science Center for Cancer Control Equity
MGH: Massachusetts General Hospital
SD: Standard deviation
